# CD44 isoforms are heterogeneously expressed in breast cancer and correlate with tumor subtypes and cancer stem cell markers

**DOI:** 10.1186/1471-2407-11-418

**Published:** 2011-09-29

**Authors:** Eleonor Olsson, Gabriella Honeth, Pär-Ola Bendahl, Lao H Saal, Sofia Gruvberger-Saal, Markus Ringnér, Johan Vallon-Christersson, Göran Jönsson, Karolina Holm, Kristina Lövgren, Mårten Fernö, Dorthe Grabau, Åke Borg, Cecilia Hegardt

**Affiliations:** 1 Department of Oncology, Clinical Sciences, Lund University, Lund, Sweden; 2 CREATE Health Strategic Center for Translational Cancer Research, Lund University, Lund, Sweden; 3 Lund Strategic Research Center for Stem Cell Biology and Cell Therapy, Lund University, Sweden; 4 Department of Pathology, Lund University Hospital, Lund, Sweden; 5 Department of Research Oncology, Division of Cancer Studies, King's College London, London, UK

## Abstract

**Background:**

The CD44 cell adhesion molecule is aberrantly expressed in many breast tumors and has been implicated in the metastatic process as well as in the putative cancer stem cell (CSC) compartment. We aimed to investigate potential associations between alternatively spliced isoforms of CD44 and CSCs as well as to various breast cancer biomarkers and molecular subtypes.

**Methods:**

We used q-RT-PCR and exon-exon spanning assays to analyze the expression of four alternatively spliced CD44 isoforms as well as the total expression of CD44 in 187 breast tumors and 13 cell lines. ALDH1 protein expression was determined by IHC on TMA.

**Results:**

Breast cancer cell lines showed a heterogeneous expression pattern of the CD44 isoforms, which shifted considerably when cells were grown as mammospheres. Tumors characterized as positive for the CD44^+^/CD24*^- ^*phenotype by immunohistochemistry were associated to all isoforms except the CD44 standard (CD44S) isoform, which lacks all variant exons. Conversely, tumors with strong expression of the CSC marker ALDH1 had elevated expression of CD44S. A high expression of the CD44v2-v10 isoform, which retain all variant exons, was correlated to positive steroid receptor status, low proliferation and luminal A subtype. The CD44v3-v10 isoform showed similar correlations, while high expression of CD44v8-v10 was correlated to positive EGFR, negative/low HER2 status and basal-like subtype. High expression of CD44S was associated with strong HER2 staining and also a subgroup of basal-like tumors. Unsupervised hierarchical cluster analysis of CD44 isoform expression data divided tumors into four main clusters, which showed significant correlations to molecular subtypes and differences in 10-year overall survival.

**Conclusions:**

We demonstrate that individual CD44 isoforms can be associated to different breast cancer subtypes and clinical markers such as HER2, ER and PgR, which suggests involvement of CD44 splice variants in specific oncogenic signaling pathways. Efforts to link CD44 to CSCs and tumor progression should consider the expression of various CD44 isoforms.

## Background

Breast cancer is characterized by a remarkable biological heterogeneity both between and within tumors. Breast tumors can be stratified into molecular subtypes using gene expression profiling [1-3] and within a tumor, a variety of cell populations with different phenotypes can be found. Earlier studies have identified a subpopulation of putative cancer stem cells (CSCs) with the phenotype CD44^+^/CD24^-/low ^[4] and more recently, aldehyde dehydrogenase (ALDH) activity was shown to mark normal as well as malignant mammary stem cells [5]. These CSCs have been associated with enhanced invasive properties [6], increased resistance to radio- and chemotherapy [7,8], as well as poorer prognosis [5,9]. Presence of CD44^+^/CD24^- ^tumor cells has also been associated with the aggressive basal-like molecular subtype of breast cancer [10].

CD44 is a transmembrane glycoprotein that participates in many cellular processes including regulation of cell division, survival, migration, and adhesion [11] through the binding of its major ligand, hyaluronic acid, and by acting as a cellular platform for growth factors and heparan-sulphate proteoglycans. It can also act as a co-receptor to mediate signaling of the HER family and MET receptor tyrosine kinases, possibly by organizing the assembly of functional complexes [12,13]. CD44 also provides a link between the plasma membrane and the actin cytoskeleton, modulating cellular shape and motility [12,13]. The human CD44 gene is located on chromosome 11p13 and consists of 19 coding exons of which 9, residing between constitutive exons 5 and 6, can be alternatively spliced into many different isoforms with tissue and differentiation-specific expression [12]. The standard isoform of CD44 (CD44S) contains none of the 9 variable exons, whereas the CD44v2-v10 isoform includes them all (exon v1 is not expressed in humans). The CD44v3-v10 isoform has one less exon and the CD44v8-v10 isoform includes only the last three of the variable exons. Additional isoforms formed by alternative splicing, and various posttranslational modifications further increase the heterogeneity of the CD44 protein products [12].

The CD44 molecule consists of an amino-terminal extracellular and ligand-binding domain, a membrane-proximal stem loop including the variable region, a transmembrane region, and a cytoplasmic tail that attaches to actin and ankyrin in the cytoskeleton [12]. The epitope recognized by the CD44 antibodies (clones 156-3C11 and G44-26) commonly used for isolation of CSCs is situated in the amino-terminal region of CD44 consisting of the nonvariable exons 1 to 5, indicating that all CD44 isoforms should be detected by this antibody [13]. Different isoforms of CD44 have been described to be involved in metastatic spread in different tumor forms even if the results are contradictory [14,15]. However, it has not been shown if the expression of specific CD44 isoforms is associated with CSCs or various breast cancer biomarkers and tumor subtypes. In order to investigate this further we analyzed the four CD44 isoforms described above using quantitative real-time PCR (q-RT-PCR) in a large material of breast tumors and cell lines.

## Methods

### Cell lines

The breast cell lines BT-474, HCC1428, HCC1937, MCF7, MCF10A, MDA-MB-231, MDA-MB-361, MDA-MB-436, SK-BR-3 and ZR-75-1 were obtained from American Type Culture Collection (ATCC, Mannanas, VA). JIMT-1 was purchased from the German Collection of Microorganisms and Cell Cultures (DSMZ, Braunschweig, Germany). L56Br-C1 was established at the Department of Oncology, Lund University [16] and PMC42 [17] was received through a generous gift from Dr. Anna Git at the Breast Cancer Functional Genomics Laboratory, Cancer Research UK, Cambridge Research Institute and Department of Oncology, University of Cambridge, UK. The HCC1937, MDA-MB-436, and L56Br-C1 cell lines are from *BRCA1 *germline mutation carriers [16].

### Cell culture

All cell lines were cultured under adherent conditions in RPMI1640 (Invitrogen, Carlsbad, CA) supplemented with 1 mM Na-puruvate, non-essential amino acids, 50 U/ml penicillin, 50 ng/ml streptomycin (all from Invitrogen) and 10% fetal bovine serum (FBS, Hyclone, Logan, UT). MDA-MB-361 was cultured as above except for 20% concentration of FBS. MCF7, BT-474, MDA-MB-436 and L56Br-C1 were additionally supplemented with 10 μg/ml insulin (Invitrogen). HCC1937 was supplemented with 10 μg/ml insulin and 20 ng/ml epidermal growth factor (EGF, Invitrogen). MCF10A was supplemented with 10 μg/ml of insulin, 20 ng/ml EGF, 100 ng/ml cholera toxin (Sigma, St. Louis, CO) and 500 ng/ml hydrocortisone (Sigma). To enrich for stem cell properties, cells were also cultured as non-adherent multicellular spheres (mammospheres) in Mammary Epithelial Growth Medium (Cambrex, Walkersville) including hydrocortisone, insulin and GA-1000) [18]. The media was additionally supplemented with 20 ng/ml of EGF, 20 ng/ml basic fibroblast growth factor (R&D Systems, Minneapolis, MN) and B27 (Invitrogen). Mammospheres were passaged once a week by enzymatic dissociation with Accutase (Innovative Cell Technologies, Inc., San Diego, CA) followed by mechanical dissociation by pipetting.

### Patients and tumors

Fresh frozen tumor tissue from 151 patients diagnosed with stage II primary breast cancer were obtained from the Southern Sweden Breast Cancer Group's tissue bank at the Department of Oncology, Lund University. These patients were treated with 2 or 5 years of adjuvant tamoxifen and were included in two previous randomized trials [19,20]. Included in the study were also 36 tumors from patients with germline *BRCA1 *or *BRCA2 *mutations (32 and 4 tumors, respectively). The tissue microarray used has been described earlier [10]. The study was approved by the regional ethical committee at Lund University (reg. no. LU240-01 and 2009/658), waiving the requirement for informed consent for the study.

### Biomarkers

Estrogen (ER) and progesterone receptor (PgR) status and S-phase fraction were obtained earlier from enzyme immunoassay and DNA flow cytometry, respectively [19,21]. Immunohistochemistry (IHC) on tissue microarrays (TMAs) was also previously used for staining of epidermal growth factor receptor (EGFR) (n = 69 tumors) and human epidermal growth factor receptor 2 (HER2) (n = 82 tumors) protein [22,23], and for double-staining of CD44 (Clone 156-3C11) and CD24 protein (n = 80) [10]. The proportion of CD44^+^, CD24^+ ^and CD44^+^/CD24^- ^tumor cells was used in scoring: 0 = 0% positive tumor cells, 1 = 1-10% positive cells, 2 = 11-50% positive cells, 3 = 51-75% positive cells, 4 = 76-100% positive cells. PTEN protein expression and *PIK3CA *mutational status were available for 110 and 107 of the tumors, respectively [24]. Microarray gene expression data was available for 157 of the tumors [25-27] and tumors were subclassified according to Hu et al. [3] as described [10]. Samples with Pearson correlation < 0.2 to all centroids were considered unclassified.

### ALDH1 expression

ALDH1 protein expression was determined by IHC on TMA. Antigen retrieval was achieved by placing the slides in citrate buffer (Dako S1699) (DAKO, Glostrup, Denmark) at 125°C in a 2100 Retriever (PickCell Laboratories, Amsterdam, the Netherlands) for 5 minutes and ALDH1 was detected with a mouse monoclonal primary antibody (Clone 44, BD Biosciences, San Jose, CA, USA) followed by EnVision™ on an Autostainer (DAKO). Cytoplasmic staining was recorded as negative (< 1% positive tumor cells), weakly or strongly positive. In total 210 breast tumors were analyzed, of which 73 were also analyzed for CD44 isoforms.

### Flow cytometric analysis of CD44 and CD24

Cells were washed once with phosphate-buffered saline and harvested using Accutase. Detached cells were labeled with fluorochrome-conjugated monoclonal antibodies obtained from BD Biosciences Pharmingen (San Diego, CA) against human CD44 (FITC; clone G44-26) and human CD24 (PE; clone ML5). Appropriate isotype controls were used to set the threshold for CD44 and CD24 positive cells. The labeled cells were analyzed on a FACSCalibur (BD Biosciences). Dead cells were excluded by staining with 7-aminoactinomycin D (Sigma-Aldrich, St. Louis, MO).

### RNA extraction

Approximately 100 mg of frozen tumor material was pulverized using a microdismembrator immediately followed by homogenization in Trizol reagent (Invitrogen, Carlsbad, CA) and total RNA was isolated according to manufacturer's instructions. A second round of purification was performed using the RNeasy kit (Qiagen, Hilden, Germany) according to manufacturer's instructions. The total RNA from breast cell lines was extracted using the RNeasy kit. The concentration of total RNA was measured by a ND-1000 NanoDrop spectrophotometer (NanoDrop Technologies, Wilmington, DE) and integrity of RNA was verified using the 2100 Bioanalyzer (Agilent Technologies, Palo Alto, CA).

### Gene expression analysis of cell lines

350 ng RNA per sample was amplified and biotinylated using Illumina TotalPrep 96 RNA Amplification Kit (Ambion, Austin, TX). 750 ng cRNA per sample was hybridized to Illumina Human-12 v3 Expression Bead Chip (Illumina, San Diego, CA) using Whole-Genome Expression Direct Hybridisation kit (Illumina) and scanned with the Illumina BeadArray reader according to manufacturer's instructions. The mean signal of the data was uploaded to BASE2 [28]. The data was quantile normalized in BASE2 and then exported into MeV [29]. Log2 transformation and median center was performed across genes. Gene expression data was available for a panel of 24 cell lines including all 13 cell lines selected for q-RT-PCR analysis. Like Neve et al. [30] we discovered 3 groups when performing hierarchical clustering based on the most varying genes and we also assigned cell lines to luminal, basal A or basal B. 21 of our 24 cell lines were included in Neve et al. and we obtained a similar clustering pattern using our data [30].

### Quantitative real-time PCR

Different mRNA transcripts of CD44 were obtained from The National Center for Biotechnology Information (NCBI) Reference Sequence (RefSeq) database. Inventoried exon-exon spanning TaqMan gene expression assays (Applied Biosystems, Foster City, CA) were used for detection of CD44; Hs00153304_m1 (CD44 total, NM_001001389.1, NM_001001390.1, NM_001001391.1, NM_001001392.1, NM_000610.3), Hs01081480_m1 (CD44v3-v10, NM_001001389.1), Hs01081475_m1 (CD44v8-v10, NM_001001390.1), Hs01081473_m1 (CD44S, NM_001001391.1) and Hs01075866_m1 (CD44v2-v10, NM_000610.3). The RefSeqs; NM_001202555.1, NM_001202556.1, NM_001202557.1 recently added to the NCBI database were not included in our study. Three different endogenous controls were used: GAPDH (4333764T), ACTB (Hs99999903_m1), and PUM1 (Hs00206469_m1) (Applied Biosystems). TaqMan Gene Expression Master Mix (Applied Biosystems) was used and all reactions were run in triplicates. A CAS1200 instrument and a Rotor-Gene instrument (Corbett Life Science, Sydney, Australia) was used for automated PCR setup of the q-RT-PCR reactions. The Rotor-Gene 6.0 software was used for calculations of average C_t_-values for each sample and the data was exported to Microsoft Excel for further analysis. The average C_t_-value for all three endogenous controls was calculated for each sample. To calculate the relative expression of the CD44 transcripts detected by each gene expression assay in respective sample the delta-delta C_t_-method was used [31]. Either one of the analyzed tumors or cell lines were arbitrarily set as a calibrator. The calculated relative expression values were log2-transformed.

### Western blot

Cells were lysed in RIPA buffer containing protease inhibitor (Roche, Basel, Swizerland) and mixed with 2× NuPage sample buffer. Samples were run on Bis Tris NuPage Gel 4-12% and then transferred to PVDF membranes (Invitrogen). Membranes were blocked with 5% non-fat dry-milk in Tris-buffered saline with 0.1% Tween (TBST) overnight at 4°C. Membranes were incubated with CD44 antibody (Clone 156-3C11) for 2 hours at RT followed by horse-radish-peroxidase conjugated goat anti-rabbit IgG (GE Healthcare, Chalfont, St. Giles, UK) for 1 hour at RT in 5% dry-milk with TBST. Visualization was performed using ECL Plus (GE Healthcare).

### Statistical analysis

Spearman correlation coefficient was used to assess the association between two continuous variables, Mann-Whitney test was used for two-group comparisons of continuous variables and Kruskal-Wallis test was used to compare expression of continuous variables in multiple subgroups. Fisher's exact test was used to analyze contingency tables. Cox regression analysis was used for analysis of time-to-event data and Kaplan-Meier plots to illustrate the results. Schoenfeld's test was used to test proportional hazards assumptions. All statistical analyses were carried out in Stata 10.0 (StataCorp LP, College Station, TX). All tests were two-sided and P-values < 0.05 were considered as significant.

### Cluster analysis

Hierarchical clustering using Pearson correlation with average linkage was performed in MeV 4.6.1 [29]. Log2 ratios were adjusted by median centering of samples followed by median centering of genes before clustering.

## Results

### Correlation between CD44 mRNA and protein expression in breast cell lines

**Figure 1:**
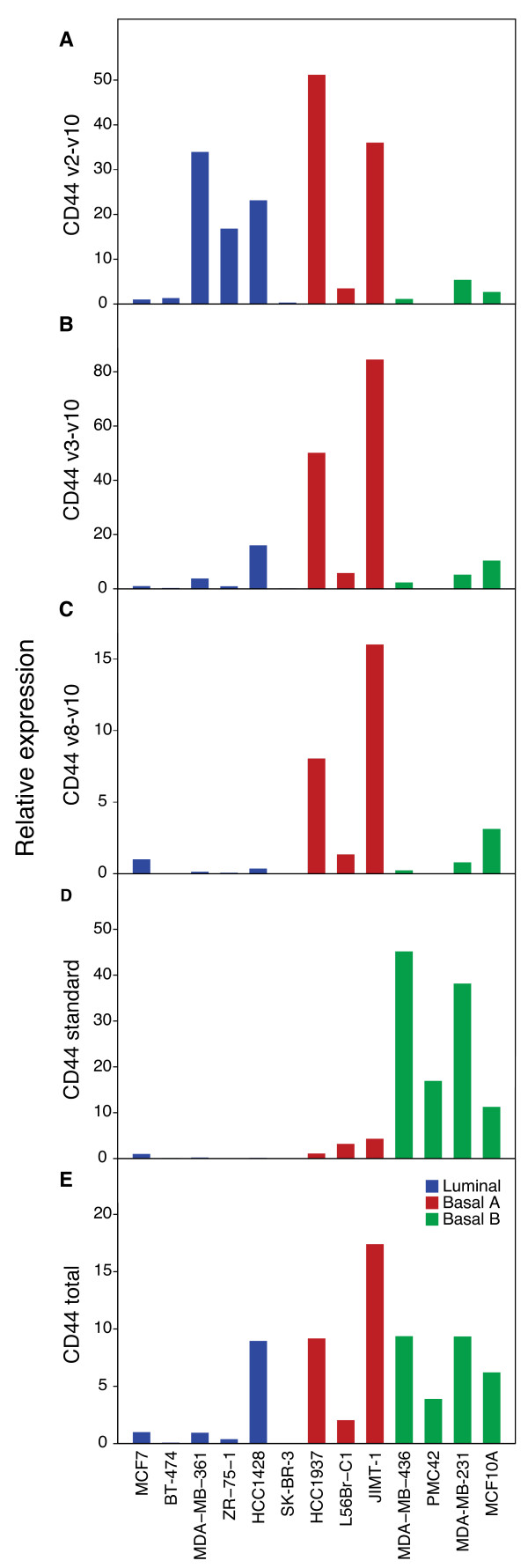
**CD44 splice variants show heterogeneous expression patterns in breast cell lines**. A to E. Q-RT-PCR analyses (on mRNA level) of the splice variants CD44v2-v10, CD44v3-v10, CD44v8-v10 and CD44S in breast cell lines of different molecular subtypes. The expression of CD44S was generally high in the basal B cell lines whereas the other isoforms were predominately expressed in the basal A cell lines.

To investigate the presence of different CD44 splice variants in breast cell lines we analyzed 13 cell lines by q-RT-PCR. The cell lines were assigned to luminal, basal A or basal B according to Neve et al [30]. The total expression of CD44 transcripts was first compared to flow cytometry data of CD44 and a relatively good agreement between mRNA and protein expression could be seen. However, the MDA-MB-361 cell line has 79.6% of CD44 positive cells despite a moderate expression level of total CD44 (Table 1). Possibly, the relatively high amount of synthesized CD44 protein in MDA-MB-361 can be explained by for example post-transcriptional modifications and translational regulation specific only for this cell line. Exon-exon spanning q-RT-PCR assays were further used to illustrate a very heterogeneous expression pattern of different alternatively spliced CD44 transcripts (Figure 1). In general, the basal A and B cell lines showed a much higher total expression of CD44 than the luminal cell lines. We also found that basal A and basal B cell lines had almost mutually exclusive expression of different CD44 transcripts. For instance, the MDA-MB-231 and HCC1937 cells had similar total CD44 protein and RNA expression levels, however, while this was primarily due to expression of CD44S in the former cells, this isoform was virtually undetectable in the latter cells. Of interest, PMC42 cells, described as having stem cell-like properties [17], showed exclusive expression of the CD44S isoform.

**Table 1:** Comparison of the proportion of CD44+ cells as determined by flow cytometry to the relative mRNA expression determined by q-RT-PCR.

Cell line	**CD44+ by FCM (% positive cells)**^**1**^	Relative mRNA expression of total CD44
MCF7	15.1	1.00
MDA-MB-361	79.6	0.95
BT-474	0	0.09
ZR-75-1	6.00	0.39
SK-BR-3	7.42	0.04
HCC1428	65.8	8.96
MDA-MB-436	97.3	9.37
L56Br-C1	49.5	2.04
MDA-MB-231	99.2	9.35
HCC1937	96.0	9.18
PMC42	98.4	3.89
JIMT-1	99.8	17.4
MCF10A	99.7	6.21

The relative mRNA expression was calculated using the cell line MCF7 as reference.

^1 ^Appropriate isotype control was used to set the threshold for CD44 positive cells.

### Altered expression of CD44 isoforms in mammosphere cultures

Four of the breast cancer cell lines (MDA-MB-231, MCF7, JIMT-1 and L56Br-C1) could be readily cultured under conditions where they form non-adherent mammospheres, a state thought to enrich for stem cell-like features [18]. Flow cytometric analysis showed that the proportion of CD44^+^/CD24^- ^cells in MDA-MB-231 was very high (> 95%) in both monolayer and non-adherent cultures while in MCF7 there was no clear increase in the initially very low CD44^+^/CD24^- ^cell proportion (< 0.3%). The CD44^+^/CD24^- ^proportion of JIMT-1 and L56Br-C1 cells increased from 45.5% to 82.6% and 1.2% to 7.2%, respectively, when grown as mammospheres compared to monolayers.

**Figure 2:**
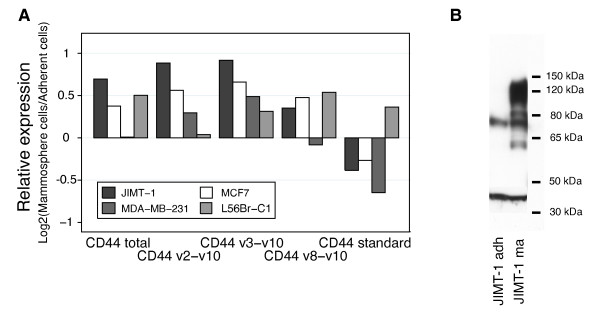
**Mammosphere propagation of cell lines changed the mRNA and protein expression of CD44 isoforms**. A. Breast cancer cell lines grown as monolayer cells or mammospheres were analyzed for mRNA expression of CD44 isoforms using q-RT-PCR. In general, the gene expression of the alternatively spliced variants CD44v2-v10, CD44v3-v10, CD44v8-v10 increased and the expression of CD44S decreased for cells cultured as mammospheres. B. Western blot analysis (CD44 antibody, clone 156-3C11) of the breast cancer cell line JIMT-1 confirmed changes in expression of CD44 variants for cells propagated as mammospheres.

We found considerable and variable changes in the expression of CD44 isoforms during these experiments (Figure 2A). Expression of CD44S decreased in three of four cell lines, the CD44v3-v10 isoform was upregulated in all four cell lines investigated and the expression of CD44v2-v10 and CD44v8-v10 were higher in mammospheres as compared to adherent cultures for three of four cell lines. For instance, MDA-MB-231 cells dramatically decreased their expression of CD44S and increased CD44v2-v10 and CD44v3-v10 expression, while the L56Br-C1 cells showed the opposite pattern. Changes in the pattern of different CD44 protein variants were also indicated by Western blot for JIMT-1 (clone 156-3C11) (Figure 2B).

### CD44 isoforms correlate to CSC biomarkers in primary breast tumors

RNA expression of CD44 isoforms was investigated in a set of 187 primary breast tumors. As shown in Table 2, the CD44v3-v10 isoform was most strongly associated to the total CD44 mRNA expression, while CD44S showed the lowest correlation. A subset of these tumors has previously [10] been stained for CD44 (and CD24) by IHC. When comparing CD44 RNA and protein expression we found that all analyzed isoforms except CD44S were positively correlated to the protein level of CD44 (Table 2). Similar results were obtained when CD44 RNA expression levels were compared with the presence of cells with the putative cancer stem cell phenotype CD44^+^/CD24^- ^(Table 3).

**Table 2:** Correlations of the mRNA expression of different CD44 splice variants to total CD44 mRNA and protein expression as determined by q-RT-PCR and IHC respectively.

Splice variant	CD44 total mRNA expression (n = 187)		CD44 protein expression (n = 80)	
	ρ^1^	P-value	ρ^1^	P-value
CD44v2-v10	0.58	**< 0.00001**	0.25	**0.026**
CD44v3-v10	0.72	**< 0.00001**	0.34	**0.002**
CD44v8-v10	0.64	**< 0.00001**	0.36	**0.001**
CD44S	0.26	**0.001**	-0.20	0.070
CD44 total	-	-	0.37	**0.001**

^1^Spearman's correlation coefficient (rho)

**Table 3:** Associations between the mRNA expression of CD44 isoforms and the cancer stem cell phenotypes CD44^+^/CD24^- ^and ALDH1^+ ^(as determined by IHC).

Splice variant	CD44+/CD24-				ALDH1+			
	All^2^ n = 80	Negative^2^ n = 51 (64%)	Positive^2^ n = 29 (36%)	P^1^	All^2^ n = 73	Neg/Weak^2^ n = 61(84%)	Strong^2^ n = 12 (16%)	P^1^
CD44v2-v10	1.650	1.580	1.843	**0.045**	1.594	1.594	1.706	0.69
CD44v3-v10	1.231	1.124	1.380	**0.002**	1.188	1.224	1.131	0.92
CD44v8-v10	1.440	1.115	1.640	**0.0007**	1.457	1.457	1.387	0.81
CD44S	0.257	0.293	0.214	**0.024**	0.261	0.240	0.467	**0.011**
CD44 total	0.702	0.628	0.818	**0.002**	0.699	0.629	0.763	0.24

^1 ^Mann-Whitney test

^2 ^Median values

**Figure 3:**
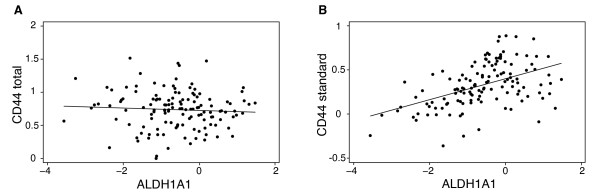
**Association between CD44 and ALDH1A1 expression in primary breast cancer**. A. Gene expression of the total CD44 shows no significant correlation to expression of ALDH1A1 (Spearman ρ = -0.10, P = 0.28; n = 131). B. The gene expression of CD44 standard isoform was positively correlated to expression of ALDH1A1 (Spearman ρ = 0.49, P < 0.00001; n = 131).

We further analyzed the correlation of CD44 isoforms to expression of ALDH1, another suggested marker of normal and malignant stem cells [5]. ALDH1 staining was detected in 31% of all 210 tumors, with a strong staining in 9%. Strong ALDH1 staining was significantly associated with negative ER (P = 0.008) and PgR status (P = 0.015), strong HER2 expression (P = 0.025) and with a high S-phase fraction (P = 0.005). ALDH1 status did not correlate with presence of CD44^+^/CD24^- ^tumor cells. We had overlapping ALDH1 IHC and CD44 q-RT-PCR data for 73 tumors, 12 (16%) of which were strongly ALDH1 positive. Surprisingly, expression of CD44S was positively associated to ALDH1+ tumors (Table 3). This was confirmed by microarray ALDH1 expression data, which showed correlation to CD44S (Spearman ρ = 0.49, P < 0.00001; n = 131) but not to other CD44 isoforms or total CD44 RNA expression (Figure 3A-B).

### Correlation of CD44 isoforms to different clinical biomarkers in tumors

The expression of different CD44 isoforms was significantly associated to certain patient and tumor characteristics (Table 4). A high expression of CD44v2-v10 was correlated to positive ER and PgR status, low S-phase fraction and postmenopausal age. High expression of CD44v3-v10 correlated to positive steroid receptor status and low proliferation, but also to negative/low HER2 status. Strong EGFR expression and negative/low HER2 status were correlated to expression of CD44v8-v10 but showed no association to steroid receptor status or proliferation rate. Interestingly, a correlation was found between the expression of CD44S and strong HER2 expression, as well as to smaller tumor size. Lymph node status could not be associated to any of the isoforms. We found a correlation of CD44v2-v10 expression to the presence of *PIK3CA *mutations. However, none of the CD44 isoforms were associated to PTEN status. 10-year overall survival was significantly better for patients with tumors showing high expression of CD44v2-v10 (HR = 0.64, 95% CI = 0.27-1.21, P = 0.007).

**Table 4:** Comparisons of the CD44 isoform mRNA levels in tumors of different clinical characteristics.

	n (%)	CD44 v2-v10^2^	P^1^	CD44 v3-v10^2^	P^1^	CD44 v8-v10^2^	P^1^	CD44 standard^2^	P^1^	CD44 total^2^	P^1^
Age	180	1.645		1.276		1.340		0.295		0.736	
< 50 years	47 (26)	1.432	**0.0063**	1.228	0.23	1.615	0.12	0.250	0.32	0.732	0.42
≥50 years	133 (74)	1.733		1.281		1.301		0.311		0.749	
Tumor size	167	1.664		1.257		1.303		0.294		0.734	
≤20 mm	46 (28)	1.791	0.22	1.311	0.48	1.547	0.41	0.398	**0.0027**	0.802	0.071
> 20 mm	121 (72)	1.638		1.248		1.258		0.271		0.720	
Lymph node status	166	1.666		1.264		1.308		0.295		0.734	
Negative (n = 0)	52 (31)	1.591	0.25	1.221	0.65	1.349	0.060	0.273	0.14	0.740	0.96
Positive (n > 0)	114 (69)	1.727		1.287		1.276		0.321		0.732	
S-phase fraction	119	1.682		1.281		1.354		0.281		0.745	
Low (< 12%)	75 (63)	1.859	**0.0001**	1.347	**0.0021**	1.322	0.86	0.283	0.92	0.802	**0.0014**
High (≥12%)	44 (37)	1.404		1.163		1.368		0.265		0.621	
ER	180	1.650		1.243		1.322		0.300		0.736	
Negative (< 25 fmol/mg)	86 (48)	1.461	**0.0020**	1.142	**0.0018**	1.440	0.32	0.309	0.53	0.706	0.086
Positive (≥25 fmol/mg)	94 (52)	1.834		1.361		1.272		0.288		0.767	
PgR	178	1.601		1.243		1.323		0.297		0.736	
Negative (< 25 fmol/mg)	107 (60)	1.562	**0.0024**	1.151	**0.0007**	1.355	0.87	0.299	0.53	0.723	0.17
Positive (≥25 fmol/mg)	71 (40)	1.875		1.461		1.303		0.293		0.771	
HER2	82	1.716		1.231		1.343		0.257		0.708	
Weak/negative	63 (77)	1.759	0.26	1.290	**0.022**	1.544	**0.014**	0.230	**0.0016**	0.737	0.054
Strong (score = 3)	19 (23)	1.594		1.139		1.115		0.442		0.682	
EGFR	69	1.664		1.237		1.312		0.254		0.712	
Weak/negative	58 (84)	1.686	0.97	1.236	0.56	1.284	**0.032**	0.257	0.34	0.708	0.43
Strong (score≥7)	11 (16)	1.580		1.237		1.734		0.202		0.818	
PTEN	110	1.675		1.276		1.347		0.269		0.730	
Negative	34 (31)	1.586	0.22	1.182	0.18	1.337	0.98	0.309	0.16	0.692	0.34
Positive	76 (69)	1.727		1.322		1.347		0.241		0.739	
*PIK3CA*	107	1.669		1.270		1.312		0.271		0.715	
Wildtype	76 (71)	1.589	**0.0036**	1.233	0.075	1.398	0.15	0.281	0.78	0.714	0.40
Mutation	31 (29)	1.965		1.355		1.178		0.249		0.752	
Distant metastasis	149	1.733		1.287		1.292		0.293		0.744	
Negative	99 (66)	1.837	0.12	1.328	0.15	1.292	0.73	0.284	0.52	0.752	0.27
Positive	50 (33)	1.618		1.199		1.294		0.304		0.726	

^1 ^Mann-Whitney test

^2 ^Median values

### Distinct expression of CD44 isoforms in tumors of different molecular subtype

**Figure 4:**
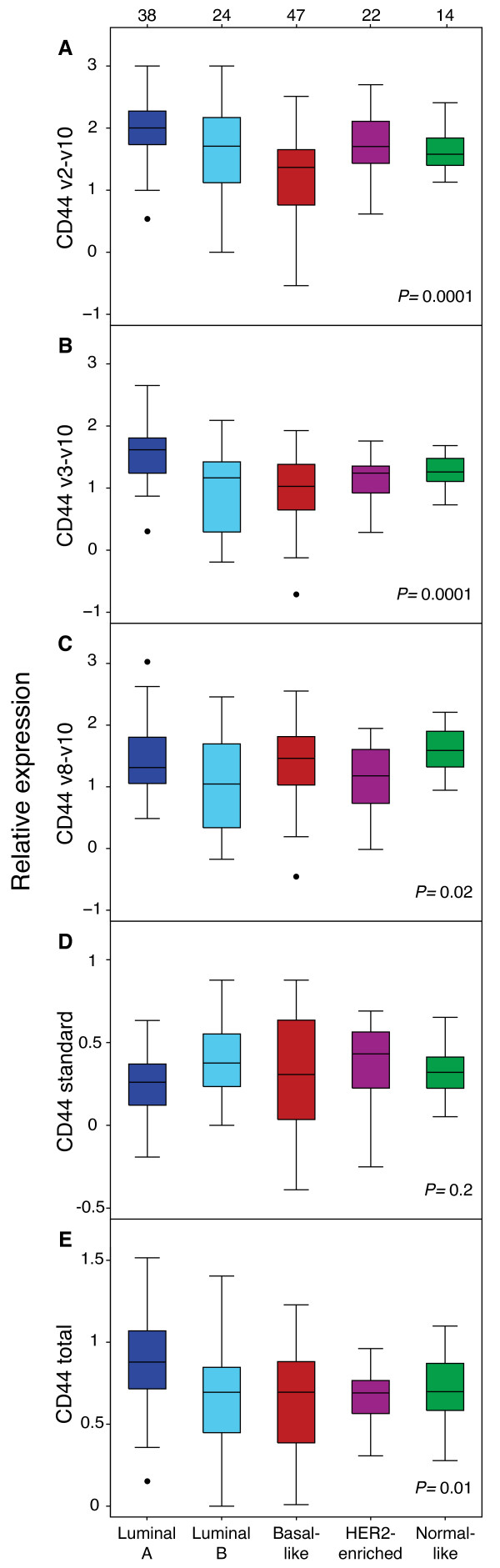
**Expression of alternatively spliced transcripts of CD44 in breast cancer tumors classified into molecular subtypes**. A-E. All isoforms except for the CD44 standard were differentially expressed in the different molecular subtypes. The CD44v2-v10 and CD44v3-v10 variants were highly expressed in luminal A tumors whereas the CD44v8-v10 isoform was associated to basal- and normal-like tumors. The CD44 standard variant had the highest median expression in HER2-enriched tumors. The numbers of tumors in each subtype are shown at top.

Gene expression microarray data was used to classify tumors into subtypes according to Hu et al. [3]. A Pearson correlation ≥ 0.2 to at least one of the five subtype gene expression centroids was found in 145 tumors, while the remaining tumors was considered as unclassified and excluded from further analysis. As expected from their predominant expression in ER/PgR positive and less proliferating tumors, the CD44v2-v10 and CD44v3-v10 isoforms were associated to the luminal A subtype and were expressed to lesser degree in basal-like tumors (Figure 4A-B). Specifically, CD44v2-v10 expression was significantly higher in luminal A compared to the basal-like (P = 0.0001), luminal B (P = 0.038), normal-like (P = 0.011) and HER2-enriched subtype (borderline significance; P = 0.051). A similar pattern was seen for CD44v3-v10 with higher expression in luminal A tumors compared to basal-like (P < 0.001), luminal B (P < 0.001), normal-like (P = 0.003) and HER2-enriched subtype tumors (P = 0.001). A different pattern was seen for CD44v8-v10, with highest expression in normal-like and basal-like tumors and lowest expression in luminal B (P = 0.018, as compared to normal-like) and HER2-enriched (P = 0.019) classified tumors (Figure 4C). The expression of CD44S varied less between subtypes but, as expected, showed highest median expression in the HER2-enriched subtype, being significant when comparing to luminal A tumors (P = 0.050) (Figure 4D). The total expression of CD44 was significantly higher in the luminal A subgroup compared to basal-like (P = 0.007), luminal B (P = 0.009) and HER2-enriched (P = 0.002) classified tumors while the normal-like subgroup showed borderline significance (P = 0.051) (Figure 4E).

The basal-like subtype showed a better 10-year overall survival for tumors with high expression of total CD44 (HR = 0.22, 95% CI = 0.06 to 0.79, P = 0.021) as well as CD44v2-v10, CD44v3-v10, CD44v8-v10 (HR = 0.44, 95% CI = 0.24 to 0.81, P = 0.008; HR = 0.29, 95% CI 0.14 to 0.61, P = 0.001; HR = 0.41, 95% CI 0.18 to 0.90, P = 0.026, respectively). Similar to basal-like tumors the luminal A subtype showed a better survival at high expression of total CD44 (HR = 0.20, 95% CI = 0.05 to 0.93, P = 0.04).

### Patterns of CD44 isoform expression in relation to biomarkers and survival

**Figure 5:**
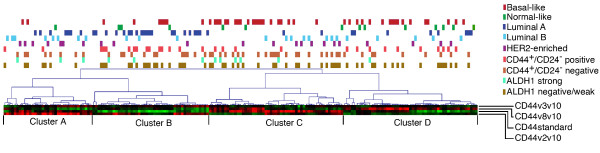
**Hierarchical clustering of breast tumors based on CD44 isoform expression resulted in four main clusters**. The heatmap shows relative mRNA expression levels (red, high; green, low). Tumors in Cluster C and D were associated to the basal-like molecular subtype and cluster B was associated to luminal A-classified tumors. Cluster B and D had the highest proportions of tumors of the CD44+/CD24- phenotype.

**Figure 6:**
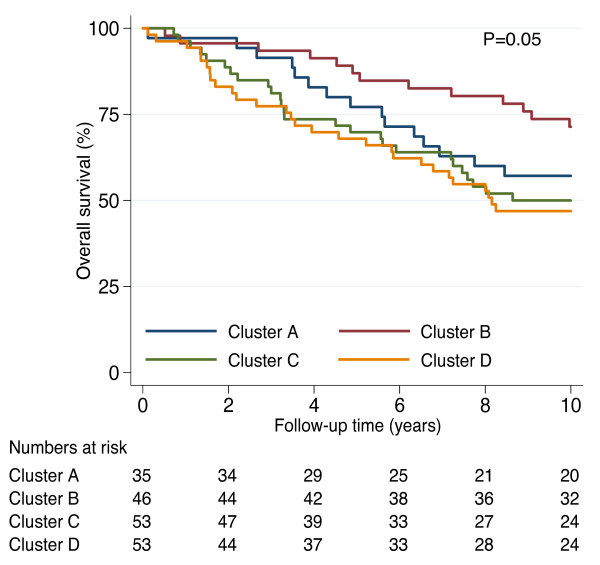
**Overall survival in patient subgroups derived from hierarchical clustering of CD44 isoform gene expression data**. The 10-year overall survival differed between the four clusters A-D (log rank P = 0.05). The number of patients at risk in each cluster is shown below the time axis.

The expression levels of CD44 isoforms were used in hierarchical clustering to subdivide the 187 tumors into four main clusters (Cluster A-D; Figure 5). Strikingly, while Cluster A had high CD44v2-v10 and low CD44v8-v10 expression, the opposite pattern was seen in Cluster D. Furthermore, Cluster B tumors showed high expression of CD44v2-v10, CD44v3-v10 and low expression of CD44S while the reverse expression pattern was observed in Cluster C. The four clusters were significantly correlated to various biomarkers (Table 5) and to 10-year overall survival (log rank P = 0.05) (Figure 6). Cluster B tumors were associated with best overall survival and was characterized by mostly ER and PgR positive status, negative/low HER2 status, low proliferation and luminal A subtype, but also by higher *PIK3CA *mutation frequency. Cluster A tumors were mostly ER and PgR positive, but also more often strongly HER2 positive and of luminal B or HER2-enriched subtypes, as well as diagnosed at higher age. Cluster A showed a slightly worse prognosis compared to Cluster B (P = 0.17, HR = 1.7, 95% CI: 0.8 to 3.6). Clusters C and D showed enrichment for the basal-like subtype and were associated to higher proliferation (Figure 5), although Cluster C included tumors with high CD44S expression and of luminal B and HER2-enriched subtype. Cluster D was enriched for tumors of both basal- and normal-like subtypes characterized by high CD44v8-v10 and low CD44S expression. Both Cluster C and Cluster D showed significantly worse prognosis compared to Cluster B (P = 0.02, HR = 2.2, 95% CI = 1.1 to 4.2; P = 0.01, HR = 2.4, 95% CI = 1.2 to 4.6, respectively). Three tumors with high CD44 gene and 11p13 amplification were all assigned to cluster D and they all had a high expression of v8-v10. Presence of tumors with the CD44^+^/CD24^- ^phenotype varied significantly (P = 0.01), being more common in Cluster B and D (Figure 5). ALDH1+ status was found in all clusters, but less common in Cluster D. Multivariate survival analysis adjusted for age, node status and tumor size showed significantly worse outcome for Cluster C and D compared to Cluster B (HR = 2.1, 95% CI = 1.1 to 4.3, P = 0.03 respective HR = 2.1, 95% CI = 1.1 to 4.4, P = 0.04), respectively, but no significant difference between Cluster A and Cluster B.

**Table 5:** Associations of cluster A, B, C and D to clinical characteristics.

	All n = 187(%)	Cluster A n = 35 (%)	Cluster B n = 46 (%)	Cluster C n = 53(%)	Cluster D n = 53(%)	P^1^
Age	180					**0.014**
< 50 years	47 (26)	3(9)	9(20)	15(30)	20(38)	
≥50 years	133 (74)	30(91)	36(80)	35(70)	32(62)	
Tumor size	167					0.601
≤20 mm	46 (28)	11(33)	13(30)	13(28)	9(20)	
> 20 mm	121 (72)	22(67)	30(70)	34(72)	35(80)	
Lymph node status	166					0.161
Negative (n = 0)	52 (31)	8(24)	10(23)	15(32)	19(44)	
Positive (n > 0)	114 (69)	25(76)	33(77)	32(68)	24(56)	
S-phase fraction	119					**0.001**
Low (< 12%)	75 (63)	19(79)	25(86)	12(43)	19(50)	
High (≥12%)	44 (37)	5(21)	4(14)	16(57)	19(50)	
ER	180					**0.009**
Negative (< 25 fmol/mg)	86 (48)	12(34)	15(33)	32(62)	27(56)	
Positive (≥25 fmol/mg)	94 (52)	23(66)	30(67)	20(38)	21(44)	
PgR	178					**0.023**
Negative (< 25 fmol/mg)	107 (60)	16(48)	20(47)	38(73)	33(66)	
Positive (≥25 fmol/mg)	71 (40)	17(52)	23(53)	14(27)	17(34)	
HER2	82					**0.012**
Weak/negative	63 (77)	8(62)	24(92)	15(60)	16(89)	
Strong (score = 3)	19 (23)	5(38)	2(8)	10(40)	2(11)	
EGFR	69					0.188
Weak/negative	58 (84)	10(100)	15(79)	20(91)	13(72)	
Strong (score≥7)	11 (16)	0(0)	4(21)	2(9)	5(28)	
PTEN	110					0.511
Negative	34 (31)	5(26)	7(23)	12(40)	10(33)	
Positive	76 (69)	14(74)	24(77)	18(60)	20(67)	
*PIK3CA*	107					**0.015**
Wildtype	76 (71)	10(56)	16(55)	26(87)	24(80)	
Mutation	31 (29)	8(44)	13(45)	4(13)	6(20)	
Distant metastasis	149					0.269
Negative	99 (66)	22(67)	32(78)	23(59)	22(61)	
Positive	50 (33)	11(33)	9(22)	16(51)	14(39)	

Unsupervised hierarchical clustering using all four isoforms of CD44 was performed to obtain the different clusters.

^1 ^Fisher's exact test

## Discussion

The CD44 molecule and several of its isoforms have attained a lot of focus during the last decades, being described as aberrantly expressed in cancer cells and involved in metastatic spread in various tumor forms [12]. However, survival data coupled to expression of CD44 isoforms have often given rise to contradictory results [14,15]. Possibly this may be due to poor specificity of different CD44 antibodies, but also to small sample numbers and biased selection, particularly in breast cancer which is a heterogeneous disease consisting of several subtypes with different biology and clinical outcome [2]. The interest for CD44 was reinforced by the finding that tumorigenicity of breast cancer cells was limited to a putative CSC subpopulation with CD44^+^/CD24^-/low ^expression [4]. Moreover, the CD44^+^/CD24^- ^phenotype was found to correlate with the more aggressive basal-like subtype of breast cancer [10]. While the concept of CSCs is still under debate and the establishment of relevant assays and markers to describe their identity ongoing, this is clearly a clinically important subject where drugs that selectively target and kill the core of tumors are being developed [32,33].

Here we present data to suggest that the analysis and study of CD44 expression in cancer development should take the presence of various isoforms into account. Since the antibody used in most previous studies for selection of CSC properties recognizes an epitope located in a non-variable region of CD44, it cannot distinguish between isoforms. This should also be the case for the studies referred to as using an anti-CD44S antibody [14]. We used q-RT-PCR with exon-exon spanning primers specific for CD44S, v2-v10, v3-v10 and v8-v10 to analyze a large set of breast cell lines and tumors.

We found a very heterogeneous expression pattern of the CD44 isoforms in the different cell lines and interestingly the basal B (also referred to as mesenchymal) cell lines show a higher expression of CD44S compared to basal A cell lines. Interestingly, a shift in splicing pattern was observed when changing from adherent to mammosphere culture and overall the expression of CD44S decreased and the other isoforms increased their expression. This illustrates a plasticity of CD44 isoform expression possibly dependent on growth conditions, which might be assigned to *in vivo *tumor growth as well.

In the patient material tumors containing cells of the CD44^+^/CD24^- ^phenotype were positively correlated to all variants except for CD44S. Instead, tumors with a strong positive staining for another CSC marker, ALDH1, were associated with CD44S expression. We found little overlap between tumors of CD44^+^/CD24^- ^and ALDH1 positive phenotype, which might indicate that these different markers symbolizes CSCs of different origin.

CD44 isoform expression data and unsupervised hierarchical cluster analysis subdivided tumors into four main groups with profoundly different isoform expression patterns. The nonrandom occurrence of tumors of particular phenotypes in the different clusters suggests that CD44 may be part of the tumor progression program that drives development to distinct molecular subtypes or, alternatively, a consequence of this process. It has been suggested that alternative splicing of CD44 is regulated by tissue-specific factors, mitogenic signals, and cell differentiation [12]. Mesenchymal cells mostly splice out the variable exons, which may explain the predominant expression of CD44S in Cluster C tumors dominated by a basal-like and more undifferentiated phenotype. Recently, a high expression of CD44S has also been shown to be essential for cells to undergo epithelial-to mesenchymal transition [34]. On the other hand, steroid receptor positive and mostly luminal tumors of Cluster A or B, with more active growth factor receptor-RAS-MAPK signaling, are more likely to retain the variable exons of the CD44 precursor transcript. The variable region encode the extracellular and membrane-proximal stem structure and a unique motif required for addition of heparan sulphate is located in exon v3, which is present in CD44v2-v10 and v3-v10 isoforms. It has also been suggested that various CD44 isoforms function as co-receptors to growth factor receptor tyrosine kinases, and/or that CD44 molecules act as platforms for matrix metalloproteinase activity and growth factor precursor cleavage [12]. Our results may suggest that EGFR signaling preferentially cooperates with CD44v8-v10, while CD44S is more advantageous in tumors with strong expression of HER2. However, since we base our analysis on nucleic acid extracts from homogenized tumor tissue, including a mixture of tumor and stromal cells, we cannot exclude the possibility that the different patterns of CD44 isoforms are influenced by the varying presence of infiltrating cells. Moreover, our assumptions depend on CD44 transcripts being translated and expressed as functional protein molecules.

Nevertheless, since CD44 has been shown to be amplified and overexpressed in breast cancer, this implicates a functional role in tumor development and growth [35]. Our results support this very well since we find associations between different CD44 splice variants and important clinical markers such as HER2, ER and PgR and also to different molecular subtypes and overall survival. Since CD44 molecules can act as co-receptors, as well as give rise to downstream signaling in many different ways, our findings are of importance in future development of therapy against CSCs.

## Conclusions

Our results suggest that specific CD44 isoforms may have distinct roles in different breast cancer subtypes and can potentially be involved in specific oncogenic signaling pathways. Attempts to link CD44 to CSCs and tumor development should consider the expression of various CD44 isoforms.

## Abbreviations

ACTB: actin, beta; ALDH: aldehyde dehydrogenase; BRCA1: breast cancer 1, early onset; BRCA2: breast cancer 2, early onset; CD44S: CD44 standard; CSC: cancer stem cell; EGF: epidermal growth factor; EGFR: epidermal growth factor receptor; ER: estrogen receptor; FBS: fetal bovine serum; FCM: flow cytometry; GAPDH: glyceraldehyde-3-phosphate dehydrogenase; HER2: human epidermal growth factor receptor 2; IHC: immunohistochemistry; MET: met proto-oncogene; PIK3CA: phosphoinositide-3-kinase, catalytic, alpha polypeptide; PgR: progesterone receptor; PTEN: phosphatase and tensin homolog protein; PUM1: pumilio homolog 1; TBST: Tris-buffered saline with Tween; TMA: tissue microarray; q-RT-PCR: quantitative real-time PCR.

## Competing interests

The authors declare that they have no competing interests.

## Authors' contributions

EO, ÅB and CH designed the study. EO and GH carried out mammosphere propagation and flow cytometric analyses. EO performed q-RT-PCR gene expression and Western blot analyses. GH, KL, MF and DG participated in the TMA IHC analyses. EO, LS, SGS, MR, JVC, GJ, KH and CH performed microarray analyses. LS and KH carried out the mutation screening. EO and PB performed statistical analyses. EO wrote the manuscript together with ÅB and CH. All authors read and approved the final manuscript.

## Pre-publication history

The pre-publication history for this paper can be accessed here:

http://www.biomedcentral.com/1471-2407/11/418/prepub
